# Early detection of postoperative acute kidney injury by Doppler renal resistive index in major lung and cardiac operations

**DOI:** 10.1186/cc13555

**Published:** 2014-03-17

**Authors:** M Arslantas, I Cinel, A Kararmaz

**Affiliations:** 1 Marmara University Pendik Education and Research Hospital, Istanbul, Turkey

## Introduction

Lung and cardiac operations cause significant changes in the fluid balance, and thus have a high incidence of development of postoperative acute kidney injury (AKI). The renal resistive index (RRI) calculated by the pattern of the renal artery flow is an indicator of renal artery flow. In this research work, we aimed to evaluate the efficiency of the RRI on the early prediction of postoperative kidney injury in major lung and cardiac operations.

## Methods

Twenty-two patients who have undergone lung or cardiac surgery were included in the study. After the kidneys were localized by ultrasonography, the best regions of blood flow were detected using color Doppler and then the arterial waveforms of these regions were obtained and optimized by Doppler. The measurements taken from three different regions were averaged. The RRI was calculated at the preoperative and postoperative first and 24th hours respectively.

## Results

A significant correlation was established between RRI and postoperative creatinine levels (*P *< 0.01). RRI values reached their highest point at the postoperative first day whereas the creatinine levels reached their highest level at the postoperative third day. Although there was no correlation between postoperative creatinine level and duration of staying in hospital, a significant relationship was detected between duration of staying in ICU and the creatinine levels (*P *< 0.01). When the cases were divided into two groups as RRI is less (*n *= 13) and larger (*n *= 9) than 0.7, significant differences were present with regard to age and creatinine levels.

## Conclusion

The RRI, which is used to evaluate renal arterial flow, is directly related with increasing renal vascular resistance in the case of AKI. The usefulness of RRI for prediction of AKI was shown both clinically following a renal allograft and experimentally in the acute tubular necrosis modeling [1],[2]. Also in septic patients this was asserted, as RRI is a better marker than cystatin C, which is one of the popular markers of recent times for prediction of development of AKI [3]. Our results show that RRI could be a simple, non-invasive and useful technique for early diagnosis of AKI in patients undergoing major operations such as lung and cardiac surgeries.
